# Effects of High-Frequency rTMS on Negative Symptoms and Cognitive Function in Hospitalized Patients With Chronic Schizophrenia: A Double-Blind, Sham-Controlled Pilot Trial

**DOI:** 10.3389/fpsyt.2021.736094

**Published:** 2021-09-03

**Authors:** Na Wen, Lei Chen, Xuemeng Miao, Min Zhang, Yaoyao Zhang, Jie Liu, Yao Xu, Siyu Tong, Wei Tang, Mengpu Wang, Jiahong Liu, Siyao Zhou, Xinyu Fang, Ke Zhao

**Affiliations:** ^1^The Affiliated Kangning Hospital of Wenzhou Medical University, Wenzhou Medical University, Wenzhou, China; ^2^School of Mental Health, Wenzhou Medical University, Wenzhou, China; ^3^Affiliated Nanjing Brain Hospital, Nanjing Medical University, Nanjing, China; ^4^Department of Psychiatry, The Affiliated Kangning Hospital of Wenzhou Medical University, Wenzhou, China

**Keywords:** schizophrenia, negative symptoms (schizophrenia), cognitive impairment, repetitive transcranial magnetic stimulation, treatment

## Abstract

This study aimed to evaluate the efficacy of high-frequency repetitive transcranial magnetic stimulation (rTMS) over left dorsolateral pre-frontal cortex (DLPFC) in ameliorating negative symptoms and cognitive impairments in patients with chronic schizophrenia. Fifty-two patients with chronic schizophrenia were randomly assigned to two groups: active rTMS group and sham rTMS group, with existing antipsychotic drugs combined 20 sessions of 10 Hz active/sham rTMS over DLPFC (20 min/session, 5 times/week). The PANSS, RBANS, and SCWT were used to evaluate the clinical symptoms and cognitive functions of the patients. Our results indicated significant improvements in clinical symptoms (PANSS total and subscale scores) and cognitive functions (RBANS total and subscale scores, card 1 and card 3 of the SCWT test) (All *p* <0.05) after 4-week intervention both in active and sham rTMS group. Moreover, the active rTMS group showed more effective on ameliorating negative symptoms (*p* = 0.002), immediate memory (*p* = 0.016) and delayed memory (*p* = 0.047) compared to the sham group. Interestingly, PANSS negative symptom scores was negatively correlated with RBANS language scores in the real stimulation group (*p* = 0.046). The study found that the high frequency rTMS stimulation over left DLPFC as a supplement to antipsychotics may have potential benefits in improving clinical symptoms and cognitive functions in patients with chronic schizophrenia.

## Introduction

Schizophrenia is a severe and chronic mental disorder that affects ~1.0% of the global population (1). Patients with schizophrenia usually suffer from positive symptoms (i.e., delusions, hallucinations, experiences of being controlled, or Confusion of thoughts) and negative symptoms (i.e., apathy, diminished expression) (2), and may experience other symptoms such as cognitive impairments (3, 4). Compared to the general population, patients with schizophrenia have a two to three times increased risk of death. Generally, the prognosis of patients with schizophrenia is poor, with about one in seven people achieve complete remission (5). Further, according to the 2016 Global Burden of Disease Study, about 1.7% of the total global years lived with disability (YLDs) is caused by schizophrenia (6).

At present, the main treatment for schizophrenia relies on antipsychotic drugs. Antipsychotic drugs have been widely used to treat schizophrenia patients since chlorpromazine was found to uniformly alleviate positive symptoms in the 1950s. Since then, antipsychotics have been the primary treatment for schizophrenia (7–9). However, these drugs have limited effect, especially on negative symptoms and cognitive deficits (9). For example, for some schizophrenia patients, even though when the positive symptoms are controlled with effective antipsychotic drugs, the negative symptoms can persist (10). Moreover, negative symptoms and cognitive deficits are common in patients with chronic schizophrenia. Psychosocial therapy may be effective for the positive and negative symptoms or cognitive symptoms of early schizophrenia, but its therapeutic efficacy may be reduced when the course of schizophrenia is prolonged (11). Therefore, it is necessary to find other treatment options, such as other non-pharmaco-therapies, to better treat schizophrenia and meet the unmet needs of patients (12, 13).

In recent years, repetitive transcranial magnetic stimulation (rTMS), a non-invasive and safe brain stimulation technology, has been widely used in the clinical treatment of mental disorders, such as schizophrenia, major depressive disorder, anxiety and insomnia (14). rTMS is based on the principle that rapidly changing magnetic field can induce electric currents in localized areas of the cerebral cortex, thereby including changes in neuronal activity in the cerebral cortex. Generally speaking, high-frequency rTMS increases cortical excitability, while low-frequency rTMS can suppress cortical excitability (15–17). In addition, rTMS can alter the metabolic activity of the brain, neuronal plasticity, local brain function, and the functional connections between different brain regions (18). rTMS may be a useful treatment for some of the symptoms of schizophrenia, such as persistent auditory hallucinations, negative symptoms (19), and cognitive impairments. The current study focused on the refractory symptoms that cannot be effectively controlled by antipsychotic drugs, including negative symptoms and cognitive function deficits. Evidences from recent studies suggest that high-frequency rTMS is an effective treatment option for improving the prognosis of schizophrenia (20), but there are mixed reports in the literature. Some studies have confirmed that high-frequency rTMS has a significant effect on negative symptoms and cognitive impairments (20–22) in schizophrenia patients. For instance, Gan et al. found that high-frequency rTMS relieved the negative symptoms (especially affective flattening and anhedonia) of schizophrenia to a certain degree and the improvement in negative symptoms lasted for at least 2 months (12). Li et al. found that an improvement in negative symptoms occurred in 8 weeks after rTMS treatment, suggesting a delayed effect of 10 Hz rTMS on negative symptoms (21). Moreover, several rTMS studies using different methods have reported beneficial effects of rTMS on single cognitive domains (i.e., working memory, facial emotion recognition, or short-term language memory) (22–24). However, other studies have reported no effect of high-frequency rTMS on clinical symptoms and cognitive impairments. For example, Wobrock et al. found that the application of active 10 Hz rTMS to the left dorsolateral pre-frontal cortex (DLPFC) was not superior to false rTMS in ameliorating the negative symptoms of schizophrenia (25). Further, Hasan et al. found that a 3-week intervention (10 Hz rTMS, 15 sessions) with active or sham rTMS produce no significant differences in negative or cognitive symptoms compared to the pre-interaction period (26). Several factors may account for the discrepancies in these studies, including the disease status of the patients (acute or stable phase) and the characteristics of the rTMS stimulation (including frequency, intensity of stimulation, and electrical placement). It should be noted that several meta-analyses with larger sample sizes have demonstrated a therapeutic effect of rTMS on negative symptoms and cognitive impairment in schizophrenia patients (27–32). These meta-analyses concluded that the best rTMS parameter for the treatment of clinical symptoms in schizophrenia is a 4-week (20 times) intervention on the left DLPFC. However, it should be noted that these previous meta-analyses did not specify the status of patients the time of study selection. Thus, effectiveness of high-frequency rTMS in patients with chronic schizophrenia remains controversial.

To this end, the aim of the present study was to determine whether high-frequency rTMS over the DLPFC (20 min/session, 5 times/week) ameliorates negative symptoms and cognitive impairments in chronic schizophrenia patients. Based on the available literature, we hypothesized that patients who received the recommended rTMS protocol may improve negative symptoms and cognitive function in patients with chronic schizophrenia.

## Methods

### Participants

Fifty-two patients were consecutively recruited into the study between December 2018 and December 2019 at the Affiliated Kangning Hospital of Wenzhou Medical University. All participants provided written informed consent and had the ability to comply with the rTMS therapy protocol and cognitive assessment. And participant's rTMS treatment fee was waived. The clinical trial protocol was approved by the institutional review committee of the Affiliated Kangning Hospital of Wenzhou Medical University.

The patients were diagnosed with schizophrenia based on the Structured Clinical Interview for DSM-IV Axis I disorders (SCID). The study inclusion criteria were as follows: (1) Han Chinese, (2) aged 18–70 years, (3) with a disease course of more than 1 year, and (4) on a stable dose of antipsychotic medication for at least 1 month before study enrollment. The exclusion criteria were: (1) any major physical diseases (e.g., cardiovascular, liver, kidney, gastrointestinal diseases, etc.), (2) the presence of a cardiac pacemaker, intracranial metal, or prior history of epilepsy or head injury, (3) female patients who were pregnant, planning to become pregnant, or breastfeeding during the study period, (4) patient had received rTMS or modified electroconvulsive therapy (MECT) in the previous month, and (5) patient had a history of alcohol or other substance abuse or dependence.

All experimental procedures in this study were carried out in strict accordance with the Declaration of Helsinki and other relevant regulations.

### Study Design

The recruited patients were assigned a sequential number. If the patient chooses to quit the study between the randomization and the rTMS intervention, this patient will be excluded from the final analyses. And then we randomly divided the patients into two groups by using default random number generator of SPSS version 25.0 (SPSS Inc., Chicago, IL). The groups were as follows: the active rTMS group (*n* = 26, with existing antipsychotic drugs + 20 sessions of 10 Hz active rTMS over the DLPFC, lasting for 20 min/session, 5 times/week) and the sham rTMS group (*n* = 26, with existing antipsychotic drugs + 20 sessions of sham rTMS over the DLPFC, lasting for 20 min/session, 5 times/week). Before the intervention, the patients didn't take any psychotherapeutic treatment. And the clinical symptoms of the two groups were basically the same at baseline. The most common antipsychotic drug taken by the patients was clozapine, followed by risperidone and olanzapine. Clinical data was collected at baseline and after rTMS treatment, including the Positive and Negative Syndrome Scale (PANSS), the Repeatable Battery for the Assessment of Neuropsychological Status (RBANS), and the Stroop Color and Word Test (SCWT). The study was a double-blind randomized control trial. The scale raters and patients were blind to the study grouping. The study was registered in the clinicaltrials.gov database (NCT04055181).

### Intervention

rTMS was administered using a YRDCCY-I stimulator (Yiruide Medical Equipment New Technology Co., Ltd., Wuhan, China) with a figure-eight-shaped coil. The patient was awake and maintained a comfortable seated position when receiving rTMS. The loop coil provides stimulation tangentially to the plane of the skull; the middle position of the loop coil is aligned with the stimulation point. Participants all received 20 treatment sessions on consecutive weekdays and were randomly assigned to receive either 10 Hz rTMS applied to the DLPFC with the YRDCCY-I stimulator or the sham condition. The rTMS was presented at 110% of the motor threshold (MT) and stimulation lasted for 4 s with 26 s intervals, with a total of 1,600 pulses per session for a total time of 20 min per day. The left DLPFC stimulation site was determined on a para-sagittal plane 5.5 cm anterior to the area of the optimal site. The sham condition involved tilting of the magnetic coil on one wing at a 45-degree angle, resulting in a similar skin sensation, but the biological activity was significantly reduced (33). Thus, in the sham group, all procedures were identical to the 10 Hz group except that in the sham rTMS, the probe of the apparatus was held perpendicular to the patient's skull plane.

### Clinical Assessments

The PANSS (34) was used to evaluate patients' psychotic symptoms. It consists of 30 items that are scored from 1 to 7, with higher scores indicating greater symptom burden. In this study, the positive (PANSS-P), negative (PANSS-N), and general psychopathology (PANSS-G) subscales as well as the total score (PANSS-T) pre- and post-rTMS treatment were analyzed.

The RBANS and the SCWT were used to assess the cognitive function in all participants. The 12-item RBANS consists of five subsets, corresponding to the following five neuropsychological processes: immediate memory, visuospatial function, language, attention, and delayed memory (35). The RBANS has good validity and reliability in Chinese people and is suitable for the cognitive evaluation of patients with schizophrenia (36). Generally, a higher RBANS score reflects a better cognitive function. The SCWT consists of three white cards containing a matrix of stimulus materials, which are words or color patches (37). The reaction time and the number of errors a participant makes when responding to the stimuli are recorded. In general, the shorter the answering time and the higher the correct rate indicate that the patient's executive function is better.

The Udvalg for Kliniske Under-sogelser (UKU) side effect rating scale was also used to evaluate side effects 4 weeks after the rTMS intervention. The scale comprises 48 items, measuring psychic, neurologic, autonomic, and other adverse effects. All scales exhibited test-retest correlations of up to 0.8 in repeated assessments (38).

All assessments were performed by at least two professionally trained psychiatrists at baseline and at 4 weeks after rTMS intervention.

### Data Analysis

Comparison of the baseline demographics and clinical features between the active and sham rTMS groups was carried out using the chi-square test for categorical variables and analysis of variance (ANOVA) for metric variables. The post-intervention data were all analyzed using repeated measures. The time course and treatment differences in relation to changes in clinical symptoms and cognitive functions were evaluated by means of a mixed-effects model for repeated measures analysis with the main effects of treatment and time and a treatment × time interaction adjusted for age, sex, education level, duration of illness, and daily antipsychotic dose. Finally, correlation analysis was carried out between the reduction in PANSS scores and improvement in RBANS in the two groups, with age, gender, education level, duration of illness, and daily antipsychotic dose as covariates. For all models, a two-sided *P*-value of < 0.05 was considered statistically significant. The statistical analyses were carried out using SPSS 26.0 (SPSS Inc., Chicago, IL, USA).

## Result

### Demographic and Clinical Characteristics at Baseline

In total, 52 patients were recruited into the study and randomly assigned to either the active rTMS (*N* = 26) or sham rTMS (*N* = 26) groups. All patients received a stable dose of antipsychotics during the treatment period. Table 1 shows that aside from the duration of illness and the negative symptoms as measured by the PANSS (both *p* < 0.05), there were no significant differences between the two groups in terms of demographic characteristics, the PANSS-T, PANSS-P, and PANSS-G, the RBANS total and subscale scores, as well as Stroop reaction time (all *p* > 0.05) at the baseline assessment.

**Table 1:** Baseline socio-demographics and clinical characteristics between groups of active rTMS and sham rTMS.

	**Active rTMS**	**Sham rTMS**	***X*^**2**^/*F***	***P***
	**(*n* = 26)**	**(*n* = 26)**		
Age (years)	41.4 ± 7.5	38.8 ± 9.1	1.27	0.26
Gender (M/F)	15/11	14/12	0.08	0.78
Education (years)	9.3 ± 3.1	8.8 ± 2.8	0.37	0.55
Age of onset (years)	23.0 ± 5.6	24.4 ± 8.5	0.48	0.49
Duration of illness (years)	18.4 ± 7.3	14.4 ± 6.5	4.37	0.04^*^
Antipsychotics type				
Clozapine	17	15	–	–
Risperidone	4	6	–	–
Olanzapine	5	5	–	–
DAD (mg)	435.8 ± 302.6	467.1 ± 267.6	0.16	0.69
PANSS total score	102.0 ± 11.2	98.7 ± 9.9	1.27	0.27
P-subscore	20.1 ± 3.7	20.5 ± 4.0	0.13	0.72
N-subscore	28.9 ± 3.9	26.7 ± 3.2	5.00	0.03^*^
G-subscore	52.6 ± 8.2	51.6 ± 7.8	0.22	0.64
RBANS total score	60.2 ± 12.1	59.3 ± 11.2	0.08	0.77
Immediate memory	51.0 ± 11.9	53.3 ± 14.2	0.40	0.53
Attention	73.4 ± 15.6	71.5 ± 16.9	0.17	0.68
Visuospatial	74.8 ± 19.7	74.9 ± 12.4	0.00	0.97
Delayed memory	63.1 ± 19.0	58.7 ± 16.4	0.79	0.38
Language	71.3 ± 15.1	68.8 ± 14.4	0.37	0.54
SCWT				
Card 1 word (s)	29.1 ± 11.4	28.2 ± 10.9	0.08	0.78
Card 2 color (s)	33.1 ± 11.8	37.0 ± 12.0	1.36	0.25
Card 3 word (s)	35.5 ± 13.3	36.5 ± 13.9	0.07	0.79
Card 3 color (s)	51.5 ± 10.0	53.7 ± 7.3	0.79	0.38

*DAD, Daily Antipsychotic Dose (chlorpromazine equivalent); PANSS, Positive and Negative Syndrome Scale; P, positive symptom; N, negative symptom; G, general psychopathology; RBANS, Repeatable Battery for the Assessment of Neuropsychological Status; SCWT, Stroop Color-Word test*.

* *p < 0.05*.

Four patients dropped out of the active rTMS group and three from the sham rTMS group during the study, leaving a final experimental sample of 22 patients in the active rTMS group and 23 patients in the sham rTMS group.

### Psychotic Symptoms and Cognitive Function After 4-week rTMS Treatment

**Figure 1:**
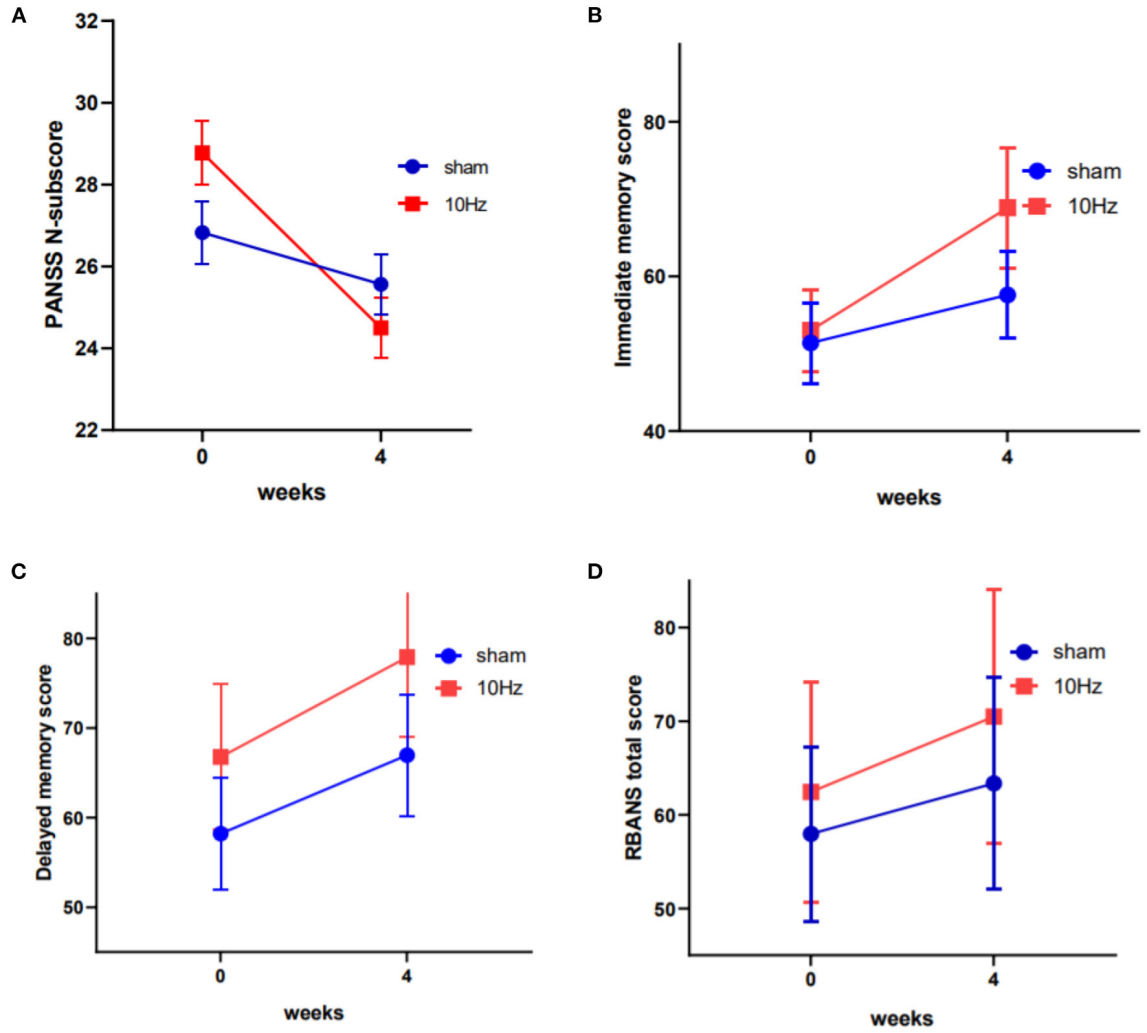
Changes in the PANSS negative symptom scores, the RBANS immediate memory scores and the RBANS delayed memory scores between active rTMS and sham group at baseline and endpoint (4th week) **(A–C)**. Changes in the total score of RBANS between the two groups was of marginal significant **(D)**.

The PANSS total and subscale scores stratified by group (active rTMS group and sham rTMS group) and time (baseline and post-treatment) are presented in Figure 1 and Table 2. The repeated-measures ANOVA showed a significant time effect (*F* = 34.9, df = 1, 43, *p* < 0.001) and an interaction effect (group × time: *F* = 10.3, df = 1, 43, *p* < 0.01), but no significant group effect (*F* = 0.21, df = 1, 43, *p* = 0.648) on negative symptoms, while there was only a significant time effect on the PANSS total score (*F* = 119.5, df = 1, 43, *p* < 0.001), positive symptoms subscore (*F* = 56.9, df = 1, 43, *p* < 0.001), and general psychopathology subscore (*F* = 29.7, df = 1, 43, *p* < 0.001). Further, ANOVA revealed that the average PANSS negative symptom score at week 4 in the active rTMS group was significantly lower than that in the sham group (*F* = 9.088, df = 1, 43, *p* < 0.01; ES = 0.327), after controlling for age, education level, duration of illness, and dose of antipsychotic drugs (chlorpromazine equivalent). However, there were no significant differences in the PANSS positive symptoms and general psychopathology scores at week 4 between the active and sham rTMS groups (both *P* > 0.05).

**Table 2:** Primary and secondary outcome measures at the beginning and the end of 4 weeks of rTMS treatment.

	**Baseline (** ***n*** **=** **52)**		**After treatment (** ***n*** **=** **45)**		**Group**	**Time**	**Group × Time**
	**Sham rTMS**	**Active rTMS**	**Sham rTMS**	**Active rTMS**	***F* (*p-*value)**	***F* (*p-*value)**	***F* (*p-*value)**
	**(*n* = 26)**	**(*n* = 26)**	**(*n* = 22)**	**(*n* = 23)**			
PANSS total score	102.0 ± 11.2	98.7 ± 9.9	86.0 ± 12.4	86.9 ± 8.8	0.78 (0.382)	119.5 (0.000)	2.2 (0.148)
P-subscore	20.1 ± 3.7	20.5 ± 4.0	16.9 ± 4.2	16.7 ± 2.8	0.03 (0.859)	56.9 (0.000)	0.64 (0.429)
N-subscore	28.9 ± 3.9	26.7 ± 3.2	25.6 ± 3.1	24.5 ± 3.9	0.21 (0.648)	34.9 (0.000)	10.3 (0.002^**^)
G-subscore	52.6 ± 8.2	51.6 ± 7.8	45.3 ± 9.6	45.6 ± 5.8	0.16 (0.690)	29.7 (0.000)	0.27 (0.607)
RBANS total score	60.2 ± 12.1	59.3 ± 11.2	63.3 ± 11.3	70.5 ± 13.5	3.0 (0.089)	62.1 (0.000)	2.4 (0.127)
Immediate memory	51.0 ± 11.9	53.3 ± 14.2	57.6 ± 13.0	68.9 ± 17.6	3.1 (0.085)	33.2 (0.000)	6.3 (0.016^*^)
Attention	73.4 ± 15.6	71.5 ± 16.9	74.7 ± 14.9	81.4 ± 13.5	1.8 (0.183)	15.6 (0.000)	0.57 (0.454)
Visuospatial	74.8 ± 19.7	74.9 ± 12.4	81.2 ± 15.2	80.4 ± 18.9	0.14 (0.707)	6.4 (0.015)	2.1 (0.155)
Delayed memory	63.1 ± 19.0	58.7 ± 16.4	67.0 ± 15.7	77.9 ± 20.1	4.2 (0.047^*^)	26.4 (0.000)	0.39 (0.538)
Language	71.3 ± 15.1	68.8 ± 14.4	70.2 ± 14.9	74.8 ± 13.0	2.0 (0.166)	3.2 (0.080)	0.41 (0.525)
SCWT							
Card 1 word (s)	29.1 ± 11.4	28.2 ± 10.9	25.1 ± 10.5	24.1 ± 10.1	0.19 (0.667)	21.8 (0.000)	0.24 (0.629)
Card 2 color (s)	33.1 ± 11.8	37.0 ± 12.0	36.2 ± 12.5	30.0 ± 9.3	3.8 (0.057)	2.0 (0.169)	0.002 (0.967)
Card 3 word (s)	35.5 ± 13.3	36.5 ± 13.9	36.6 ± 14.3	36.7 ± 10.8	0.32 (0.575)	0.50 (0.485)	1.3 (0.265)
Card 3 color (s)	51.5 ± 10.0	53.7 ± 7.3	55.5 ± 8.1	54.4 ± 7.8	0.99 (0.327)	4.9 (0.031)	1.4 (0.249)

*PANSS, Positive and Negative Syndrome Scale; P, positive symptom; N, negative symptom; G, general psychopathology; RBANS, Repeatable Battery for the Assessment of Neuropsychological Status; SCWT, Stroop Color-Word test*.

* *p < 0.05*,

** *p < 0.01*.

In terms of changes in cognitive function, the repeated-measures ANOVA showed a significant time effect (*F* = 62.1, df = 1, 43, *p* < 0.001) and a marginally significant group effect (*F* = 3.0, df = 1, 43, *p* = 0.089) on RBANS total scores; however, there was no significant interaction effect (*F* = 2.4, df = 1, 43, *p* = 0.127). Further, the RBANS subscales were analyzed with repeated-measures ANOVA and the results showed a significant time effect (*F* = 33.2, df = 1, 43, *p* < 0.001) and interaction effect (group × time: *F* = 6.3, df = 1, 43, *p* < 0.05), as well as a marginally significant group effect (*F* = 3.1, df = 1, 43, *p* = 0.085) on immediate memory, a significant time effect (*F* = 26.4, df = 1, 43, *p* < 0.001) and group effect on delayed memory, and significant time effects on attention (*F* = 15.6, df = 1, 43, *p* < 0.001) and visuospatial/constructional function (*F* = 6.4, df = 1, 43, *p* < 0.05) (see Table 2 and Figure 1). The ANOVA further indicated that immediate memory was significantly better in the active rTMS group than in the sham group at week 4 (*F* = 6.713, df = 1, 43, *p* = 0.013; ES = 0.161), after controlling for age, duration of illness, dose of antipsychotics, and PANSS negative symptoms subscore.

### Associations Between the Reduction of Negative Symptoms of Schizophrenia and the Improvement of Cognitive Function

**Figure 2:**
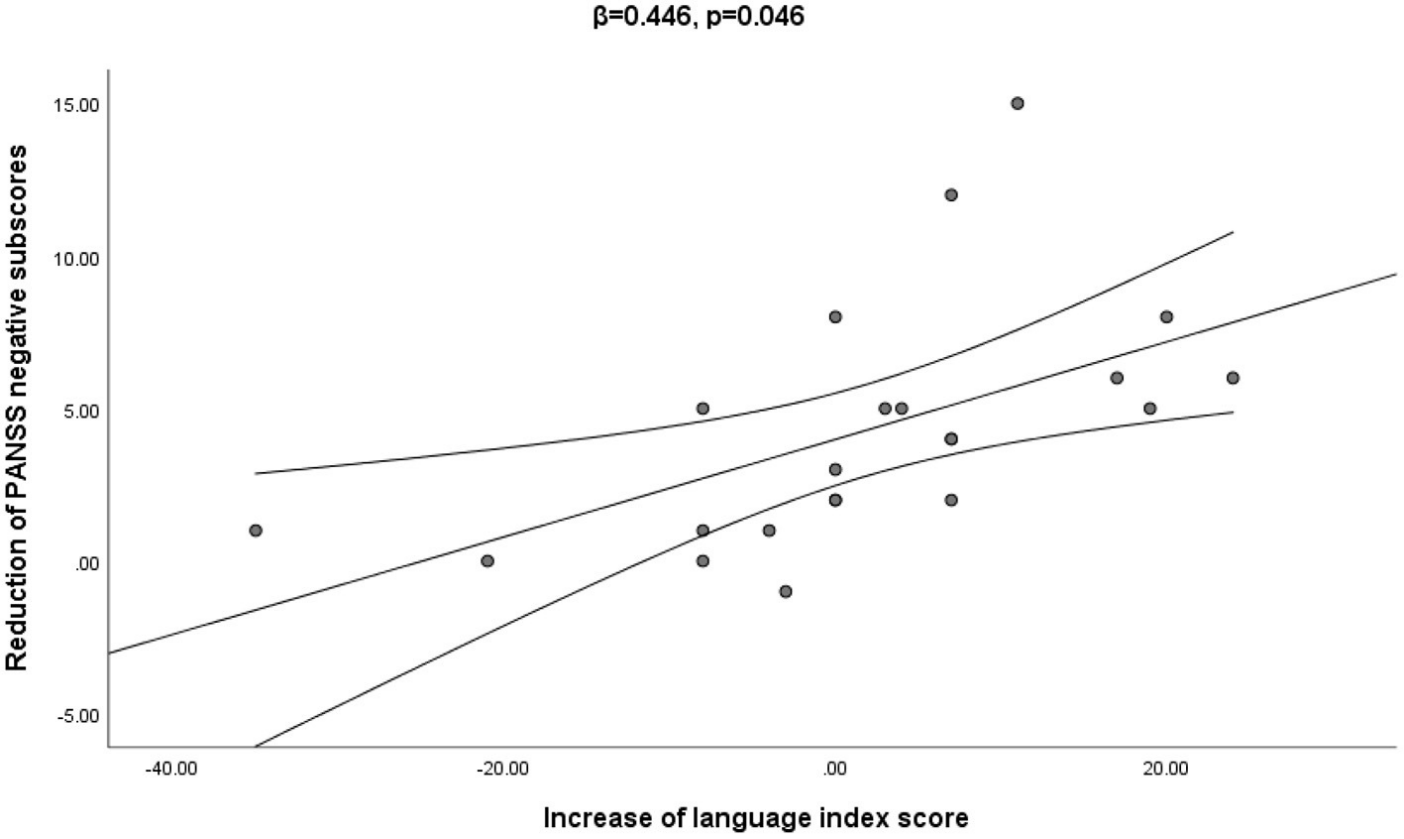
The multiple regression analysis confirmed that the increase in language index score was significantly associated with the decrease of the PANSS negative score from baseline to week 4 (β = 0.446, *p* = 0.046).

The correlation analysis showed that the change in RBANS language subscore was negatively correlated with the change in the PANSS negative subscore when age, education level, duration of illness and DAD (β = 0.446, *t* = 2.15, *p* < 0.05) were all controlled (see Figure 2).

### Safety Assessment

After 4 weeks of treatment, four patients in the active rTMS group reported mild adverse reactions (one reported a reduced duration of sleep, one reported emotional indifference, two reported tension headaches) and three patients in the sham rTMS group reported mild adverse reactions (one reported a reduced duration of sleep and two reported tension headaches). There was no significant difference in the incidence of adverse events between the two groups (*p* > 0.05).

## Discussion

As a non-pharmacological treatment strategy, rTMS has great application prospect for the treatment or cure of schizophrenia. Research has shown that rTMS may reduce positive and negative symptoms in patients with schizophrenia who take antipsychotic drugs, but there has been significant heterogeneity in the reported effects in different trials (39). In contrast to previous studies, the current study focused on exploring the role of high-frequency rTMS on both negative symptoms and cognitive deficits in chronic schizophrenia patients. The efficacy of high-frequency (10 Hz) rTMS over left DLPFC in ameliorating psychotic symptoms and cognitive impairments in chronic schizophrenia patients was evaluated. After the 4-week intervention, active rTMS was found to improve negative symptoms and immediate and delayed memory in schizophrenia patients. Further, our study found that the improvement in cognitive function in the active rTMS group was positively correlated with the decrease in negative symptoms score in hospitalized patients with chronic schizophrenia, which is consistent with the previous study (40).

Other studies had reported similar benefits of rTMS on negative symptoms in patients with schizophrenia. For example, Prikryl et al. found that high-frequency (10 Hz) rTMS stimulation of the left DLPFC with high stimulation intensity effectively reduced the negative symptoms of schizophrenia (41). Kumar et al. verified that the rTMS intervention with a frequency of 10 Hz may lead to better improvement of negative symptoms (42). Research suggests that the efficacy of rTMS on negative symptoms is best with a 10 Hz stimulating frequency and a longer stimulation period, ideally 4–6 weeks (29). Further, a recent study not only showed negative symptom improvement after 4 weeks of 10 Hz rTMS over the DLPFC, but this effect was maintained at the 24-week follow up (43). Most previous studies that treated schizophrenia patients with 20 Hz rTMS over the left DLPFC also showed significant improvements in negative symptoms (44–46). What we know is that higher frequencies of rTMS (frequencies of 5 Hz and greater) have been shown to have excitatory effects on neurons in the stimulated cortex (47). Earlier research found that schizophrenic patients exhibited hypoactivity of the pre-frontal cortex (48), which is related to the negative symptoms. By stimulating the cerebral cortex, the activity of the cortex increases, and the negative symptoms improve. Furthermore, recent research reported that disruption of the cerebellar-pre-frontal network functional connection was the basis for the negative symptoms in schizophrenia (49). The disrupted network connectivity may be restored with rTMS, resulting in a reduction in negative symptoms. Interestingly, previous human and animal studies have also indicated that rTMS induces dopamine release in the pre-frontal cortex (50, 51). Therefore, the release of endogenous dopamine in subcortical structures may be the most likely mechanism underlying the improvement in negative symptoms by rTMS. In addition, Kirschner et al. suggested that the improvement of depression would reduce negative symptoms with the reduction of secondary negative symptoms (52). We only asked the individuals whether they had depressive symptoms verbally, while we didn't conduct a scale assessment. Even though, we also speculated that rTMS may improve negative symptoms by affecting depressive symptoms. We will further verify this in future studies.

However, other studies have failed to find any benefit of 10 Hz rTMS on negative symptoms in patients with schizophrenia. Holi et al. found no significant difference in negative symptoms of schizophrenia between the group who received 10 Hz rTMS and the sham treatment group, though both groups showed improvement in negative symptoms (53). Wobrock and colleagues performed a sham-controlled, randomized multicenter trial with 76 schizophrenia patients treated with 10 Hz rTMS to the left DLPFC. The results revealed no statistically significant difference in improvement in negative symptoms between the active and sham rTMS groups at day 21 or subsequently through today 105 (25). The discrepancy in the treatment effect of 10 Hz rTMS over the DLPFC on negative symptoms among different studies may be due to complex confounding factors, such as heterogeneity in the sample, the assessment tool used for negative symptoms (54), total stimulation number or duration, number of treatment sessions, concomitant medication, sample size, and the setting of the clinical trial (25). Hence, more research should be performed to identify the optimal mode at a frequency of 10 Hz over DLPFC to achieve the best improvement effects on negative symptoms.

The current study showed a beneficial effect of 10 Hz rTMS on cognitive function, including immediate memory and delayed memory. An early study found that both 10 and 20 Hz rTMS improved memory in patients with schizophrenia, while another study found that both 10 and 20 Hz rTMS had delayed effects on cognitive function at the 6-month follow-up (55). Guan et al. also found the effectiveness of high-frequency rTMS stimulation in improving the cognitive function of patients with schizophrenia (56). A recent meta-analysis also found that 10 Hz rTMS over the DLPFC significantly improved all indicators of working memory performance, including reaction time and accuracy (57). It has been well-documented that abnormalities in beta and gamma-band activity are implicated in the cognitive deficits in schizophrenia (58). High-frequency rTMS may be a possible approach for cognitive improvement in schizophrenia patients via the modulation of gamma oscillatory activity in the brain. Interestingly, we found a significant correlation between the decrease in PANSS negative scores and the increase in RBANS language scores. Previous studies have shown that negative symptoms aggravate cognitive impairment in schizophrenia, and the current findings further highlight the significance of focusing on improving negative symptoms, which will, in turn, promote cognitive rehabilitation to a certain extent (59). The exact mechanism underlying the effect of rTMS on cognitive impairment and negative symptoms in schizophrenia remains unclear. Many studies have demonstrated that cognitive impairment and negative symptoms in schizophrenia share a common pathological mechanism, which may be associated with structural and functional abnormalities in the frontal lobe of the brain (19, 60). The improvement in immediate memory and delayed memory by active rTMS treatment over the DLPFC in chronic schizophrenia patients may be explained by the enhanced cortical excitability and metabolic activity of target neurons in the pre-frontal cortex, which is the brain area responsible for memory function. Nonetheless, this association and the mechanisms behind it deserve more research.

In the present study, there was no significant difference in the improvement in positive symptoms between the active rTMS and sham groups, which is in agreement with most previous studies (39, 61–63). The most important reasons underlying the lack of improvement in positive symptoms may be the frequencies and sites of stimulation. Evidence suggests that low-frequency (≤1 Hz) rTMS over the temporal-parietal cortex (TPC) could significantly ameliorate positive symptoms, especially in relation to auditory hallucinations (28, 62). Therefore, the improvement of positive symptoms by 10 Hz rTMS over DLPFC may not be significant. Future studies should verify this hypothesis.

The current research has several limitations: (1) the relatively small sample size meant that there was limited statistical power to detect differences between the groups; (2) the relatively short intervention period made it impossible to compare whether there was a difference between the short- and long-term effects of rTMS treatment;(3) the rTMS stimulation site was not guided by MRI;(4) there are no assessment and follow-up of depressive symptoms;(5) our study excluded patients who had received rTMS treatment more than a month before the beginning of this study, but some researchers believed that the benefits of rTMS on negative symptoms can be maintained for several months (43), so patients who had previously received rTMS treatment may have an impact on the results. We will pay attention to these issues in our future studies.

## Conclusions

The current study sheds light on the effect of high-frequency (10 Hz) rTMS over the left DLPFC on negative symptoms, immediate memory, and delayed memory in chronic schizophrenia patients. As a non-pharmacological strategy, rTMS has broad application prospects. Our study provides some practical significance to the clinic that when curing some treatment-resistant symptoms of schizophrenia, especially negative symptoms and cognitive deficit, 10 Hz rTMS maybe a good treatment.

## Data Availability Statement

The original contributions presented in the study are included in the article/supplementary files, further inquiries can be directed to the corresponding author/s.

## Ethics Statement

The studies involving human participants were reviewed and approved by the Affiliated Kangning Hospital of Wenzhou Medical University. The patients/participants provided their written informed consent to participate in this study.

## Author Contributions

KZ, XF, and SZ conceptualized and designed the study. NW, LC, MZ, YZ, XM, MW, and WT recruited the participants and completed the screening assessments. JL, YX, ST, and XF performed the rTMS manipulation. NW, LC, MZ, JL, and KZ analyzed the data and performed the statistical analysis. NW, LC, and SZ wrote the first draft of the manuscript. All authors revised the manuscript and approved the final manuscript.

## Conflict of Interest

The authors declare that the research was conducted in the absence of any commercial or financial relationships that could be construed as a potential conflict of interest.

## Publisher's Note

All claims expressed in this article are solely those of the authors and do not necessarily represent those of their affiliated organizations, or those of the publisher, the editors and the reviewers. Any product that may be evaluated in this article, or claim that may be made by its manufacturer, is not guaranteed or endorsed by the publisher.
